# Syngas generation from different types of sewage sludge using microwave-assisted pyrolysis with silicon carbide as the absorbent

**DOI:** 10.1016/j.heliyon.2023.e14165

**Published:** 2023-03-01

**Authors:** Doo Young Oh, Daegi Kim, Hanna Choi, Ki Young Park

**Affiliations:** a Department of Civil and Environmental Engineering, Konkuk University, 120 Neungdong-ro, Gwangjin-gu, Seoul 05029, Republic of Korea; b Department of Environmental Technology Engineering, Daegu University, 201 Daegudae-ro, Jillyang-eup, Gyeongsan-si, Gyeongsangbuk-do 38453, Republic of Korea; c Taeyoung E&C, 111 Yeouigongwon-ro, Yeongdeungpo-gu, Seoul 07241, Republic of Korea

**Keywords:** Sewage sludge, Microwave assisted pyrolysis, Absorber, Syngas, Hydrogen

## Abstract

In this study, the pyrolysis of sewage sludge was explored through microwave-assisted pyrolysis. Three kinds of sludge (primary sludge, waste-activated sludge, and digested sludge) from a sewage treatment process were used. All three kinds of sewage sludge had a low microwave absorption capacity; therefore, an absorber was added to enable microwave-assisted pyrolysis. By using silicon carbide as the heating element, it was possible to increase the temperature within a short time by applying microwaves. During the microwave-assisted pyrolysis of sewage sludges, the amount of gas generated and the H_2_ and CO fraction of the produced gas increased as temperature increased. The pyrolysis of waste-activated sludge produced the greatest quantity of gas. However, the primary sludge produced the highest amount of syngas in terms of H_2_ and CO, which indicate the high-quality of the syngas.

## Highlights

• Sewage sludge is a continuous and abundant biomass resource.
• The characteristics of sewage sludge vary depending on the wastewater treatment process.
• MWP with SiC was carried out in the target temperature, rapidly.
• As the MWP temperature increased, the H2 and CO in gas fraction increased.
• The cold gas efficiency was the 7.3% at 600 °C, 22.5% at 800 °C, and 41.4% at 1000 °C.

## 1 Introduction

The amount of sewage generated globally has increased because of urban development following industrialization coupled with changes in people's living standards, resulting in an increase in the need for sewage treatment facilities. Sewage sludge is of great public concern because of its potential risk to the environment and human health, and the considerably high operating costs of wastewater treatment plants (WWTP) [1].

In sewage treatment facilities, sludge with different characteristics is generated because the composition of sewage sludge changes depending on the characteristics of the treated sewage [2]. Based on the process of sewage treatment facilities, generated sewage sludge can be divided into three main types: Primary sludge (PS) generated from the primary settling tank; waste-activated sludge (WAS) generated at the end of the activated sludge process; and digested sludge (DS) generated after anaerobic digestion. Although sewage sludge has different characteristics depending on the generation process, all three kinds of sewage sludge have a composition comparable to that of plant tissue, including cellulose, hemicellulose, and lignin, with a high content of organic matter. Because of its abundance and consistent production during sewage treatment, it is also considered a useful biomass resource [3,4].

Methods for converting sewage sludge into fuel and recycling it as a biomass resource are continuously being studied. Energy recovery processes include incineration; biogas, bio-oil, and syngas production; and carbonization [5,6]. One study showed that when incineration was applied to recover energy from sewage sludge, the moisture content of the original sludge was approximately 85–95%, which considerably increased the cost of pretreatment required for drying and treatment [7]. Biogas recovery through anaerobic digestion is the most extensively used method of energy conversion using sewage sludge. Although this method has the advantage of recovering energy as methane gas with a low energy input, it generates a large amount of DS [8,9].

Pyrolysis has recently been studied as an alternative method for efficient energy conversion. It is a method for decomposing substances by applying heat under anoxic conditions, and similar to incineration, it is effective at utilizing sewage sludge [10]. However, unlike incineration, it has the advantage of reducing the emissions of substances that are harmful to the environment and the human body. Based on the conditions of product generation, thermal decomposition can be classified as carbonization, liquefaction, and gasification, and based on the reaction time, the rate of temperature increase, and reaction temperature, it can be further classified as either slow, fast, and flash thermal decomposition. Although some researchers have studied the processes of bio-oil production and carbonization, these processes have high associated conversion costs and produce a low product quality [[11], [12], [13], [14]]. Conversely, syngas is a known high-quality product, but the reaction time, reaction temperature, and rate of temperature increase are important for pyrolysis gas production, which require effective pyrolysis technology for rapid thermal decomposition.

In microwave pyrolysis, the temperature increases from the inside of the material, resulting in a faster heating time than that in conventional pyrolysis, which transfers heat from the surface of the material [8]. In addition, microwave pyrolysis is advantageous for fast and flash pyrolysis applications owing to its high-power density, fast internal heating, and efficient heat transfer [[15], [16], [17], [18]]. However, the microwave absorption capacity of materials is important for microwave pyrolysis. The microwave absorption capacity can be measured and compared using the electric loss tangent factor (tan δ), which can be expressed as a dielectric constant (ε′) and a dielectric loss (ε’’). The dielectric constant refers to the polarization capacity by an electric field and dielectric loss refers to the efficiency with which electromagnetic energy can be converted into heat. The loss tangent factor (tan δ = ε’’/ε’) describes the ratio of dielectric loss to dielectric constant [19].

The loss tangent factor of sewage sludge ranges from 0.1 to 0.52, and can vary depending on the conditions [18,[20], [21], [22]]. Therefore, an absorber is necessary to absorb microwaves. Graphite, activated carbon, and silicon carbide (SiC) have been used as representative absorbers. Among them, SiC has attracted attention as a high-temperature structural material because of its excellent high-temperature strength and suitability for high-temperature applications such as pyrolysis [23]. It is necessary to optimize the microwave-assisted pyrolysis conditions to maintain a high temperature when using an absorber.

The purpose of this study was to investigate the characteristics of microwave assisted pyrolysis with SiC as a heating element according to the type of sewage sludge. Moreover, we identified the optimal pyrolysis conditions for high H_2_ and CO fraction.

## 2 Materials and methods

### 2.1 Sewage sludge

In this study, three different kinds of sewage sludge were collected from the J-WWTP in Seoul, South Korea: PS was generated from the first settling tank of the WWTP, WAS was generated from the secondary settling tank of the biological treatment process, and DS remained after anaerobic digestion. Table 1 shows the characteristics of the three kinds of sewage sludge including proximate, ultimate, and inorganic property analyses used in this study. As compared to woody biomass, sewage sludge has a higher ash content, and lower fixed carbon and volatile contents [24]. However, the nitrogen and hydrogen contents tend to be slightly higher in sewage sludge than in woody biomass. The main sources of nitrogen are proteins and peptides [25]. The sewage sludge used in the present experiment had a relatively high volatile matter content and low carbon content [[26], [27], [28], [29]].

**Table 1:** Characteristics of sewage sludge.

		PS	WAS	DS
Proximate analysis	Moisture (%)	97.54	99.11	97.19
	V.M. (wt. %)^a^	74.68	71.79	54.61
	F.C. (wt. %)^a^	4.43	1.27	0.4
	Ash (wt. %)^a^	20.89	26.94	45.39
Ultimate analysis	C (wt. %)^a^	33.38	43.66	28.94
	H (wt. %)^a^	5.47	6.21	4.71
	N (wt. %)^a^	5.81	4.62	3.77
	S (wt. %)^a^	0.68	0.63	1.29
	O (wt. %)^a^	33.77	17.94	15.9
Low heating value (kcal/kg)^a^		3388.36	4307.41	2861.26

V.M.: volatile matter, F.C.: fixed carbon.

a On dry basis.

### 2.2 Microwave pyrolysis unit

**Fig. 1:**
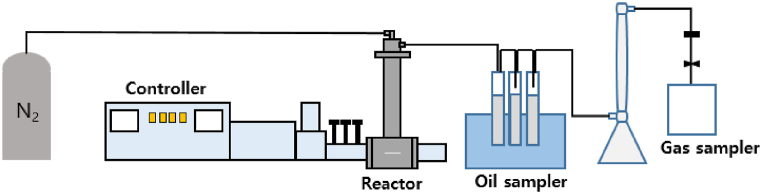
Microwave pyrolysis system.

As shown in Fig. 1, the microwave-assisted pyrolysis (MWP) system (2450 MHz microwave system, Korea Microwave Instrument Co., Korea) used in the experiment consisted of an MWP reactor, an oil recovery device, and a gas recovery device. The high-frequency output device can operate at a frequency of 2450 MHz and a maximum power of 1 kW, and the outputs can be adjusted by adjusting the target temperature. The interior of the reactor section consisted of a quartz tube. The solid product that remained inside the reactor after the experiment was recovered. Oil was recovered from the back of the reactor in three 100 mL recovery devices using dichloromethane as an organic solvent. The generated gas was collected in a 5 L gas Tedlar gas sampling bag and analyzed for gas composition.

### 2.3 Experimental method

The pyrolysis temperature conditions were 600, 800, and 1000 °C, with a reaction time of 30 min at the final temperature. The power required to reach the reaction temperature was less than 0.4 kW, and after reaching the final temperature, less than 0.2 kW was required. Nitrogen gas was pumped at a rate of 50 mL/min to maintain anaerobic conditions and to simultaneously serve as a carrier gas. In the case of thermal decomposition using microwaves, it is difficult to increase and maintain the target temperature when only the experimental sample is used. To compensate for this, a heat absorber was used. Sludge samples with a weight of 5 g each were mixed with the same amount of the heating element, and then loaded into the reactor. In this experiment, SiC was used to generate and maintain heat as the microwave absorber and heating element, respectively. The used heating elements as SiC had a relatively high inorganic SiO_2_ content of 97.57% and a carbon content of 23.63% [8].

### 2.4 Analytical procedures

Microwave pyrolysis converts sewage sludge into solid, liquid, and gaseous products. The recovered outputs were used to calculate the recovery. The solid sample was pulverized to a particle size of 100 μm for the analysis. Elemental analysis was performed using a Flash 2000 Elemental Analyzer, and the low heating value (LHV) was analyzed using a Parr Model 1341 plain jacket calorimeter. Inorganic analysis was performed using a Smartlab multi-purpose X-ray diffractometer. Thermogravimetric analysis (TGA) was performed using a TG 209 instrument (Netzsch Instruments). The gas product, i.e., synthesis gas, was analyzed with a gas chromatograph equipped with a thermal conductivity detector (GC-TCD, 6500 GC system, YL Instruments). Two types of columns (Supelco stock #13052-U and #13047-U) were used. The components of analyzed gas were: H_2_, CO, CO_2_, CH_4_, C_2_H_4_, and C_2_H_6_.

## 3 Results and discussion

### 3.1 Characteristics of sludges

The composition of sewage sludge, which varies depending on the type of sewage, is an important factor for determining the thermochemical conversion characteristics of the fuel. Table 2 shows the organic and inorganic properties of the sewage sludge. The organic composition of the three kinds of sludge is somewhat different, but most of them have a low lipid content and are mostly composed of carbohydrates and proteins.

**Table 2:** Organic and inorganic contents in the sludges.

		PS	WAS	DS
Organic content (wt. %)^a^	Carbohydrate	44.86	38.28	50.67
	Lipid	5.76	4.94	0.09
	Protein	41.30	39.86	45.46
	Fiber	8.07	16.92	3.78
Inorganic content (wt. %)^a^	Al_2_O_3_	17.42	11.36	24.11
	K_2_O	3.69	4.45	2.36
	CaO	12.27	20.79	9.12
	MnO	0.14	0.17	0.24
	Fe_2_O_3_	7.85	7.92	13.38
	CuO	0.13	0.19	0.29
	ZnO	0.37	0.48	1.00
	MgO	1.54	1.64	1.27
	SiO_2_	35.56	24.89	20.91
	TiO_2_	1.16	1.43	0.83
	other	19.85	26.68	26.49

a On dry basis.

The inorganic components present in sewage sludge play an important role in pyrolysis as Fe, K, Mg, and Ca promote secondary reactions during thermal decomposition. Table 2 shows the comparisons of organic and inorganic contents in the sewage sludges. The Fe_2_O_3_, CaO, K_2_O, and MgO contents in the three kinds of sewage sludge were compared, and it was observed that the Fe_2_O_3_ content in DS (13.38%) was higher than that in both PS (7.85%) and WAS (7.92%). Moreover, the CaO content in WAS (20.79%) was higher than that in WAS and DS, and the K_2_O content in DS (2.36%) was lower than that in PS and WAS. The three kinds of sludge showed similar results for MgO.

Table 3 shows the analysis of lignocellulose contents in the sewage sludges. Although there was a difference in content, the three kinds of sludge had the highest lignin content, followed by hemicellulose, and the cellulose content was very low.

**Table 3:** Lignocellulosic contents in the sludges.

	PS	WAS	DS
Lignin (wt. %)^a^	24.54	21.27	23.39
Cellulose (wt. %)^a^	4.2	12.88	1.87
Hemicellulose (wt. %)^a^	12.17	12.65	13.42

a On dry ash-free.

**Fig. 2:**
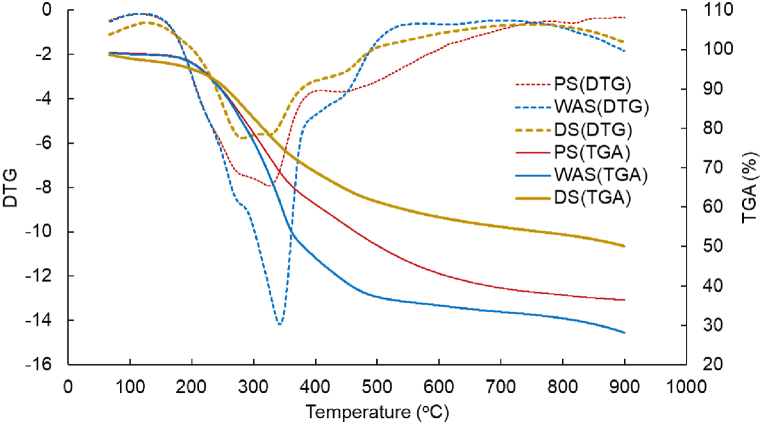
Thermogravimetric analysis of sewage sludge.

Fig. 2 shows the results of the TGA for the pyrolysis of sewage sludge. The observed weight loss between 100 and 200 °C was because of the evaporation of water in the sludge and was almost the same in all three kinds of sludge. A rapid weight loss was observed at 250 °C, which is considered to be due to the biological decomposition of organic matter [27]. The weight loss of WAS was the greatest in this step, followed by that of PS and DS. This may be because of the differences in carbohydrate, lipid, and protein contents in the three kinds of sludge. There was rapid weight loss at a temperature of approximately 500 °C, after which the weight decrease became insignificant. This is because of the difference in the decomposition temperature range of carbohydrates, lipids, and proteins, with carbohydrates decomposing at 255 °C, lipids at 300 °C, and proteins at 360–525 °C [26]. The further weight loss at temperatures above 500 °C may be explained by the decomposition of substances such as lignin and cellulose which are more difficult to decompose as in Table 3 [27].

### 3.2 MWP of sewage sludge

**Fig. 3:**
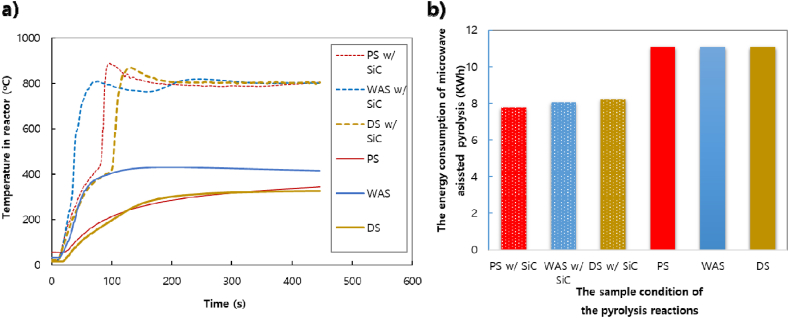
Reactor temperature curve of sewage sludge by microwave irradiation with/without SiC; a) the reactor temperature curve and b) energy consumption.

MWP was carried out in the temperature range of 600–1000 °C. The temperature rises rapidly, and accordingly, the time required to increase the temperature can be shortened significantly. In the case of MWP, it is difficult to raise and maintain a high temperature without a heating element. As shown in Fig. 3 a), SiC was used to ensure the stability of the temperature increase and its maintenance at a high value. Regarding the energy consumption, Fig. 3 b) shows that the MWP with SiC can save 27% more energy than the MWP without SiC. Previous research [8] has also shown that the energy consumption of MWP with SiC was much lower than that of conventional pyrolysis. SiC can be used as a heating element because of its high-temperature strength and excellent properties, such as abrasion, oxidation, and corrosion resistance [21]. As for a sludge as a small tan δ, it is difficult to increase the target temperature even if sufficient microwaves are irradiated [8,18]. Although moisture is the most commonly used heat absorber, it was difficult to raise the temperature using only water-containing sewage sludge. In this study, the SiC was used to both generate and maintain heat, and this method had a large tan δ heating element of microwave heating.

**Fig. 4:**
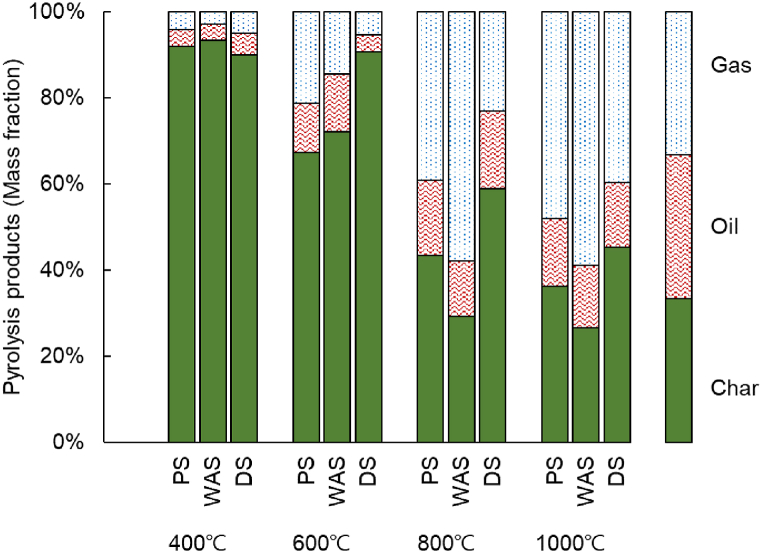
Microwave-assisted pyrolysis product mass balance for the three kinds of sewage sludge.

Fig. 4 shows the change in the composition ratio for recovered products according to the type of sewage sludge and pyrolysis temperature. In all three kinds of sludge, the gas production fraction increased, and the oil and char production fraction decreased with an increase in the pyrolysis temperature. The oil production fraction of PS and DS increased by up to 18% at 800 °C, and those of WAS did not show any significant changes in the study temperature range of 600–1000 °C. The components in WAS decomposed at 800 °C, producing 58–59% gas and 13–14% oil. This is likely because of the decomposition of a high content of proteins, fats, and carbohydrates through thermal decomposition, as shown in the thermogravimetric analysis results in Fig. 2. For PS and DS, it was confirmed that pyrolysis gas proceeded at a high temperature of 1000 °C or more. DS showed the lowest gas recovery among the three types of sludge. DS has a high ash content and is composed of materials that are relatively difficult to decompose compared to those in PS or WAS. Therefore, the fraction of char production was higher for DS than that for PS and WAS, even when the decomposition temperature increased.

### 3.3 Gas products from sewage sludge pyrolysis

**Fig. 5:**
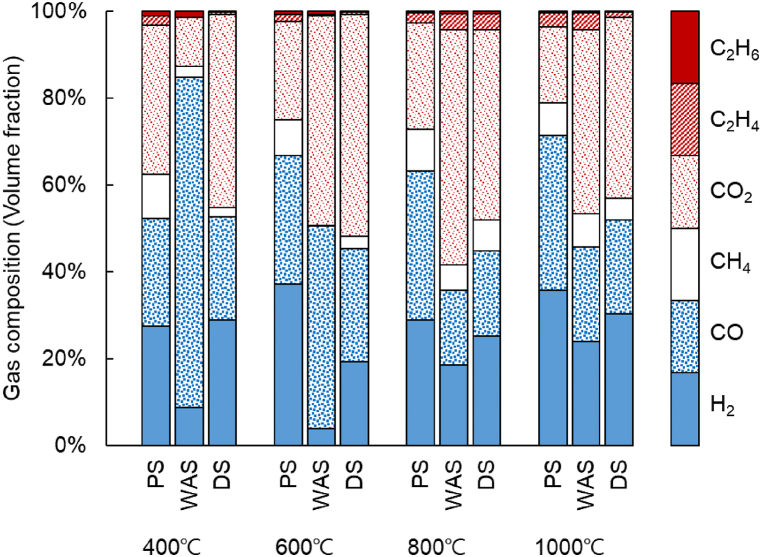
Composition of the gases from the microwave-assisted pyrolysis of the sewage sludges.

The thermogravimetric analysis confirmed that MWP actively occurred as the temperature increased, and the amount of gas produced increased with increasing temperature. Fig. 5 shows the composition of the gases produced by the pyrolysis of the three kinds of sewage sludge. The H_2_ and CO contents produced by the pyrolysis of PS were 31.49–38.14% and 31.78–38.08%, respectively, higher than those from the other sludges. Compared with the pyrolysis of PS, the H_2_ and CO fraction increased sharply at 800 °C, reaching 18.53% and 23.91% at 1000 °C, respectively. In the case of DS, the H_2_ fraction was 25.18% at 800 °C and 30.28% at 1000 °C. However, the produced CO_2_ fraction was 42.46–54.24% for WAS and 41.49–51.00% for DS, which was higher than that for PS (18.83–26.68%). In general, the H_2_ and CO contents increased, while the CH_4_ and CO_2_ contents decreased with an increasing temperature. This can be explained by the gas generation process for the thermal decomposition of organic matter [18].

**Fig. 6:**
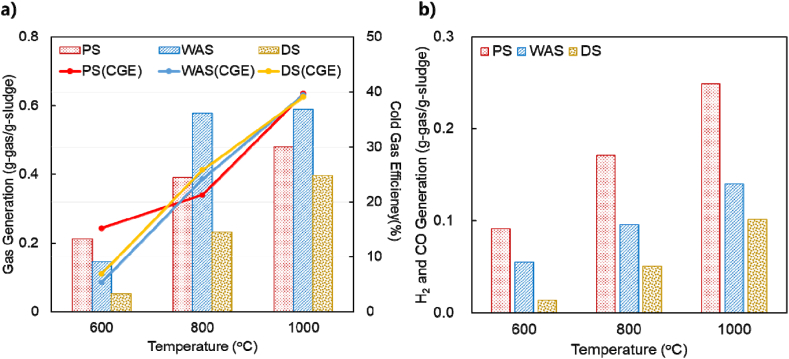
Pyrolysis gas production of sewage sludge; a) Total gas with CGE, and b) H_2_ + CO.

Fig. 6 compares the relative gas generation and composition according to the type of sewage sludge. As shown in Fig. 6 a), the greatest amount of gas was generated by WAS, at 0.58 g-gas/g-sludge at 800 °C and 0.59 g-gas/g-sludge at 1000 °C, increasing rapidly from the decomposition temperature of 600 °C, with similar results at 800–1000 °C. For the pyrolysis of DS, the amount of gas generated increased rapidly with increasing decomposition temperature, with the production of 0.05 g-gas/g-sludge at 600 °C, 0.23 g-gas/g-sludge at 800 °C, and 0.40 g-gas/g-sludge at 1000 °C. PS pyrolysis produced 0.21 g-gas/g-sludge at 600 °C, which was the highest among all sludges. Moreover, PS pyrolysis produced 0.39 g-gas/g-sludge at 800 °C and 0.48 g-gas/g-sludge at 1000 °C, higher than those for DS pyrolysis and lower than those for WAS pyrolysis. Fig. 6 b) shows the results of the comparison of gas production from the three kinds of sludges, focusing on the amounts of H_2_ and CO produced.

Among the three sludges, although WAS underwent thermal decomposition at the lowest temperature of 600 °C, it can be used directly as a fuel in the MWP process. The H_2_ and CO contents are important for the application of synthetic gas to turbines for energy generation [30]. The production of high-quality gas with high H_2_ and CO contents is an essential element for the pyrolysis gas. The gas production was the highest for WAS, whereas the total H_2_ and CO contents were the highest for PS. The total H_2_ and CO produced by PS pyrolysis were 0.14 g-gas/g-sludge at 600 °C, 0.27 g-gas/g-sludge at 800 °C, and 0.37 g-gas/g-sludge at 1000 °C. The WAS, with the highest gas production, produced the following contents of H_2_ and CO: 0.07 g-gas/g-sludge at 600 °C, 0.21 g-gas/g-sludge at 800 °C, and 0.27 g-gas/g-sludge at 1000 °C. When PS was pyrolyzed at 1000 °C, it was confirmed that more than 76% of the total gas produced was H_2_ and CO. This result is similar to that for previous sludge pyrolysis process including the gasification studies that showed high syngas yields because of the high emission of volatiles, endothermic reactions, and tar reforming and cracking during high-temperature pyrolysis [31,32]. The pyrolysis of DS produced the lowest amounts of H_2_ and CO, with 0.02 g-gas/g-sludge at 600 °C, 0.10 g-gas/g-sludge at 800 °C, and 0.21 g-gas/g-sludge at 1000 °C. This seems to be because of the low carbon and high ash contents in DS than those in PS and WAS. DS has low carbon and high ash contents because carbon-based organic matter is discharged as biogas through biological decomposition during anaerobic digestion.

The results show that the cold gas efficiency (CGE) increased the pyrolysis reaction temperatures increased (shown in Fig. 6 a)). The CGE was calculated by equations (1), (2), as follows:

(1) $$CGE\left(\%\right)=\frac{Y\times \left(H_{2}\%+{CH}_{4}\%+CO\%\right)\times {LHV}_{gas}}{M\times L{HV}_{feed}}$$

where Y is gas yield (Nm^3^), M is feed weight (g), LHV_feed_ is the low heating value of feed (kcal/kg), LHV_gas_ is the low heating value of gas(kcal/L) [33].

(2) $${LHV}_{gas}=\left(0.258\times H_{2}\%+0.0302\times co\%+0.0856\times {CH}_{4}\%+0.1432\times C_{2}H_{4}\%+0.1523\times C_{2}H_{6}\%\left(kcal/L\right)\right)$$

where *LHV*_*th*_ is the theoretical low heating value of gas on 2580 kcal/Nm^3^ of H_2_, 3035 kcal/Nm^3^ of CO, 5860 kcal/Nm^3^ of CH_4_, 14,320 kcal/Nm^3^ of C_2_H_4_, and 15,230 kcal/Nm^3^ of C_2_H_6_ [33]. Units were converted according to the formula.

Therefore, when the MWP reaction temperature increased, the CGE of all sludges increased. The higher the CGE. The CGE of PS and WAS pyrolysis were 15.2% and 5.4% at 600 °C, 21.2% and 24.1% at 800 °C, and 39.6% and 39.5% at 1000 °C, respectively. The DS, with the highest CGE, produced the following 6.9% at 600 °C, 25.8% at 800 °C, and 39.1% at 1000 °C.

## 4 Conclusion

The number of sewage treatment facilities has increased because of urbanization and the improvement of living standards, which in turn has increased the amount of generated sewage sludge. Sewage sludge contains a large amount of organic matter and is an abundant biomass resource that can be used continuously. The characteristics of sewage sludge vary depending on the treatment process. In this study, the characteristics of three kinds of sewage sludge generated in the sewage treatment process were identified. Moreover, the composition of their pyrolysis products was analyzed through the thermal decomposition of each sludge. Microwaves were used to generate heat for pyrolysis reaction at temperatures of 600, 800, and 1000 °C, because they lead to a faster increase in temperature than conventional heating methods. Sewage sludge has a low microwave absorption capacity, so SiC was used as a heat absorber to both increase and stabilize the temperature. By using SiC as an absorber, the temperature of all three kinds of sewage sludge could be rapidly increased and maintained at the target temperature for 30 min. As the pyrolysis temperature increased, the produced gas fraction increased, and the fraction of oil and char decreased. The pyrolysis of WAS generated the largest amount of gas. The greatest amount of high-quality gas (H_2_+CO) for energy production was produced during the pyrolysis of PS. In this study, a general trend of increasing H_2_ and CO contents and decreasing CH_4_ and CO_2_ contents was observed with increasing temperature. DS showed the highest increase of CGE from 6.9% at 600 °C to 39.10% at 1000 °C. At a 1000 °C of MWP, the CGE of all sludges was similar at 39.1–39.6%.

## Author contribution statement

Doo Young Oh: Performed the experiments; Analyzed and interpreted the data; Contributed reagents, materials, analysis tools or data; Wrote the paper.

Daegi Kim: Conceived and designed the experiments; Analyzed and interpreted the data; Contributed reagents, materials, analysis tools or data; Wrote the paper.

Hanna Choi: Conceived and designed the experiments; Performed the experiments; Contributed reagents, materials, analysis tools or data; Wrote the paper.

Ki Young Park, PhD: Conceived and designed the experiments; Contributed reagents, materials, analysis tools or data; Wrote the paper.

## Funding statement

Professor Ki Young Park was supported by National Research Foundation of Korea (NRF-2021R1A2C2011536), Korea Environmental Industry and Technology Institute (2021002690005) and by 10.13039/100018324Korea Environment Corporation through Waste to Energy-Recycling Human Resource Development Project (YL-WE-21-001).

## Data availability statement

Data included in article/supp. material/referenced in article.

## Declaration of interest’s statement

The authors declare no competing interests.
